# Dental Dispensaries as Sanitary Educators of the Public

**Published:** 1910-02-15

**Authors:** Carl R. Lindstrom

**Affiliations:** Boston, Mass.


### DENTAL DISPENSARIES AS SANITARY EDUCATORS OF THE PUBLIC.

BY CARL R. LINDSTROM, D.D.S., BOSTON, MASS.

Two words, now gaining standing in the vocabulary of the common people, as well as in the working speech of social reformers, stand for great constructive ideas and ideals which are to reign during this century. These words are Prevention and Co-ordination. Clergymen as well as chemists, educators as well as bacteriologists, philanthropists as well as physicians are bent upon preventing, where formerly they were keen to remedy. The time of attack is earlier, the stage of intervention is a more fundamental one, the intention is to conserve, not merely preserve or repair. Hence the altered program of many a church, school and altruistic agency. Hence the emergence in the realm of state and national activity of new policies, designed to prevent economic waste, to conserve the natural wealth.

Coincident with this universal trend towards constructive and conserving social action, goes the other and natural movement for co-ordination of remedial and preventive agencies. The reason is simple. Waste in fighting against waste, is illogical. Duplication of machinery and effort in doing common work is absurd. Moreover, where there still remains justification for separate existence and specialized functioning, the interdependence of all life, makes co-operation necessary.

The dentist of to-day and to-morrow faces a world of this sort. The call of society upon him, to serve it as promoter of sound physical health, as a teacher of oral hygiene to schools and social settlements, as a factor in human uplift, is coming to be as strong, as it hitherto has been upon men of other professions. And this service he is to render best when co-operating with educators, sanitarians, scientifically trained administrators of charity, physicians, and disseminators of intelligence like journalists and authors.

A dental dispensary and clinic, carried on with the ideals of Prevention and Co-ordination in mind, must furnish, as its range of activity extends, and its prestige waxes, a centre of information valuable not only to the dental profession, but to all the other social agencies. The visitor of the Lynn Dispensary, for instance, through her personal observation of children's homes, and their environment in other respects, is competent to advise with such other social workers as the city may have, on general social problems. Seeing many of the children repeatedly, she has noted their defects physical and mental, which may be remedied in part or in whole, by co-operative treatment of dentist, physician, surgeon or alienist, providing the discoveries made by dentist and visitor in the dispensary are followed up by co-ordinated social action. Co-operating with school authorities, and they with dental dispensaries, an attack can be made at once on conditions, which repeated examinations have made clear, exist in all schools of all classes of society, and which account for so much of the unsatisfactory results of our public and private schools. The dispensary dentist and the visitor, not only may, but do get, through the contract which the service makes possible, information as to malnutrition, impaired breathing capacity and general physical conditions, which they can at once use for the benefits of patients and society at large, in a way impossible at present with the private patient. Moreover, it is not beyond the range of possibility that the dispensary visitor in supplementing the work of the dentist, as she or he goes from home to home, may detect conditions there, which, when made known in suitable ways to proper authorities, would be abolished in the interests of morality and sound social health. Beginning with simple care of the teeth, the work in its larger ramifications may come to be a factor in civic reform.

In fact, both theory and practice, as the four month's experiment at the Lynn Dispensary has shown, point clearly to the intimate relation between dental sanitation and sanitation in general. The process of combating ignorance or defiance of the one leads to light on the problem of the other.

The best proof of the service rendered by a dental dispensary augmented by its intelligent sympathetic visitor is a concrete case of betterment of total physical and mental condition. One of the first cases brought in by the visitor for treatment at the Lynn Dispensary was a child about eleven years old. She was anaemic, hollow-eyed, gaunt, palpably underfed and diseased. Examination of the mouth showed that every tooth in her mouth was decayed. Some were so far gone as to be beyond treatment, all needed attention. The net impression of the mouth was one of utter neglect and general decay. Pus exuded from several of the sockets, and the total effect was revolting to the observer.

The task of cleansing, repairing, filling and extraction completed, the child was preached to in all seriousness as to her future duty, and equipped with brush and powder, was sent home, but not forgotten. The visitor immediately visited the home and impressed on child and parents the duty of change of habits and constant care. Close watch was kept on the child. Immediately a change for the better in physical appearance was noticeable. It has continued to this day so that the transformation is startling. Freed from pain at night, the child sleeps. She chews her food, because the chewing apparatus is in order. The masticated mass enters the stomach and intestines minus numerous bacilli, which formerly were present. Consequently, the child has gained in flesh, in color, in animation, and in zest in life.

Nor is this *all*. She has become a zealous, youthful missionary of the gospel which has saved her. She has won her own family of four persons, to a care of their teeth never known before. She has converted her little playmates, back to better habits in dental hygiene at home, and also to resort to the dispensary for examination of their teeth and such care as is prescribed for them there. This gospel she preaches insistently, when at play or as a regular business of street corner propaganda. And it is an effective sort of preaching, because so wholly unselfish, and because so splendidly supported by the transformation in her own case.

The case is especially interesting because of the extremity of her need, and the ardor of her zeal in streading the "good news" of physical redemption; but it is a *typical* case, not an extreme or singular one at all. Thousands of children in the schools, for lack of similar investigation, are the easier prey to disease, are slow and lethargic in study and "retard" the class. Co-operating in similar service, schools, settlements and dental dispensaries, can work out transformations in physical, mental and economic efficiency which will add vastly to the wealth of the country.

It may be of interest to some of you to know a little more about this Lynn Dispensary, as I consider it a fair illustration of the phase of usefulness we are trying to extend.

Owing to the generous co-operation of eleven Lynn men and some seven or eight from Boston and vicinity, we have been able to treat 175 children, representing 500 operations. The work is two-fold, consisting of dental operations accompanied by instruction in hygiene of the mouth, and the necessity of proper mastication; and secondly, of following-up work in the homes of the patients. The children who have come to us represent six nationalities.

Through the courtesy of both the Florence Manufacturing Company and the Theodore Metcalf firm we are able to buy a good brush at 50 cents a dozen and powder of simple ingredients at 25 cents a pound. These we sell at actual cost.

These are "good things" which we are trying to "push along," but of course their adoption is a slow process. We meet with strenuous opposition to our preaching an individual brush and powder for each one. One little girl was quite willing to own them for her own needs, when told how necessary to cleanliness and health but she "*did not* think *papa* needed one, for he had one he had used since he was ten years old, though the hairs *had* come out *some*, and it *was* one-sided."

But we have conquered in about 100 cases and the reform is slowly but surely *spreading*.

The work in its second phase of visits to the home has been carried on from the beginning, by the secretary of the Dispensary. About 200 calls have been made during this period of four months. It is the purpose of these calls to impress upon the parents—the mother usually—the absolute necessity of caring for the teeth by the habitual use of the brush after each meal, to urge her to co-operate with the dentist in his efforts to put the child's mouth in a normal condition. The number of calls is not limited to the number of applicants as some cases need following up repeatedly.

In order to best acquaint the City of Lynn with the establishment of the Dispensary, it has been the desire of those interested and in charge, to reach such children as need the help, through the schools by examination by teachers and school physicians, and reference to the clinic of such cases as cannot afford to employ the regular practitioner, although our rule is to make a small charge for all services rendered, whenever practicable; also by five or ten minute talks on Oral Hygiene as well as the necessity of thorough mastication, by the secretary in each class room, at stated intervals of time. It is, therefore, with great pleasure that we report the recent endorsement and active co-operation of the School Board of Lynn to take effect in the school work this coming fall. This opens a path of assured success for the work, by the official recognition and support of school trustees, through whose co-operation *should be found* the natural and legitimate door to prosperity.

Our work is meeting with assured recognition from the larger body also. We have the official endorsement of the Secretary of the State Board of Charities, of the Secretary of the Society for the Control and Relief of Tuberculosis, and of the Secretary of the State Board of Education. —*The Journal.*

